# Supplementary material for: Awake suppression after brief exposure to a familiar stimulus
Source: Commun Biol. 2021 Mar 17;4:348. doi: 10.1038/s42003-021-01863-2 (PMC7969731; doi:10.1038/s42003-021-01863-2)
Supplement: Supplementary file 2 — Supplementary Information [file 42003_2021_1863_MOESM2_ESM.pdf]

Supplementary Information

Awake suppression after brief exposure to a familiar stimulus

Ji Won Bang[1,2*], Dobromir Rahnev[1]

[1]School of Psychology, Georgia Institute of Technology, Atlanta, GA, 30332, USA

[2]Department of Ophthalmology, School of Medicine, New York University, New York, NY, 10016, USA

*Corresponding author: JiWon.Bang@nyulangone.org

Supplementary Figures 1-5

Supplementary Table 1

Supplementary Fig. 1. Threshold S/N for the familiar (trained) and novel (untrained) orientations before and after training.

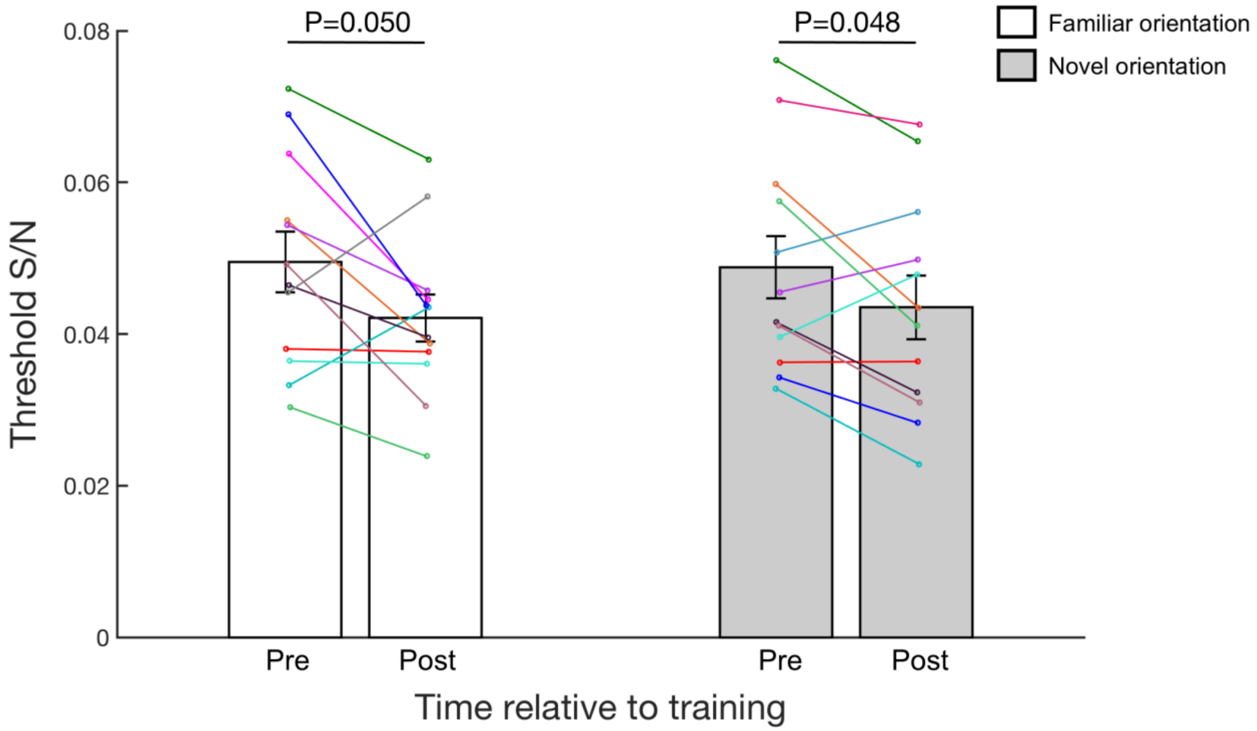

To examine if training made subjects better on the task, we performed a two-way repeated measures ANOVA with factors time (pre vs. post) and orientation (trained vs. untrained orientation). The results showed a significant main effect of time ($F(1,11)=7.441$, $P=0.020$, partial $\eta^2=0.404$), suggesting that training improved subjects' performance. The learning amount did not differ between the familiar (trained) and novel (untrained) orientations (interaction between time and orientation: $F(1,11)=0.336$, $P=0.574$, partial $\eta^2=0.030$) and was significant for each orientation (familiar orientation: $T(11)=2.199$, $P=0.050$, Hedges' $g=0.556$; novel orientation: $T(11)=2.230$, $P=0.048$, Hedges' $g=0.343$; paired t-tests). Error bars indicate s.e.m. Dots indicate individual data. N=12.

Supplementary Fig. 2. Pattern similarity for the familiar orientation before and after exposure to the familiar orientation.

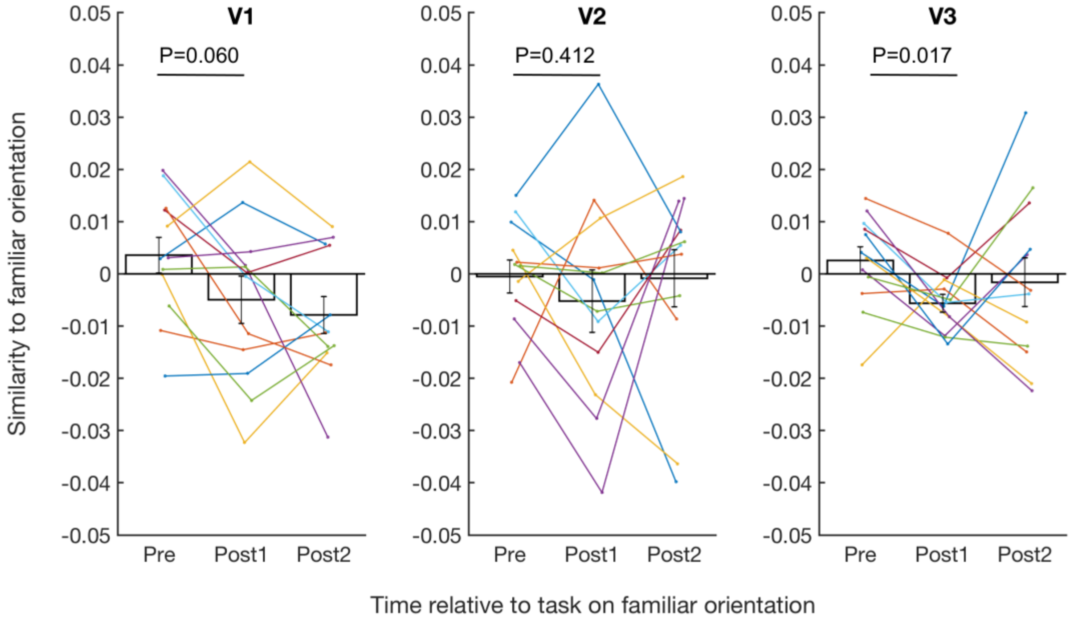

Brain activity was less similar to the familiar orientation across V1-V3 shortly after exposure to the familiar orientation, consistent with the presence of awake suppression. ‘Pre’ refers to two initial scans before subjects saw any stimuli (5 min/scan; combined into a single ‘Pre’ baseline). ‘Post1’ and ‘Post2’ refer to the first and the second post-task scans immediately after exposure to the familiar orientation. The P values in the figure refer to the results of paired t-tests between pre and post1. Error bars indicate s.e.m. N=12.

Supplementary Fig. 3. Pattern similarity for the novel orientation before and after exposure to the familiar orientation.

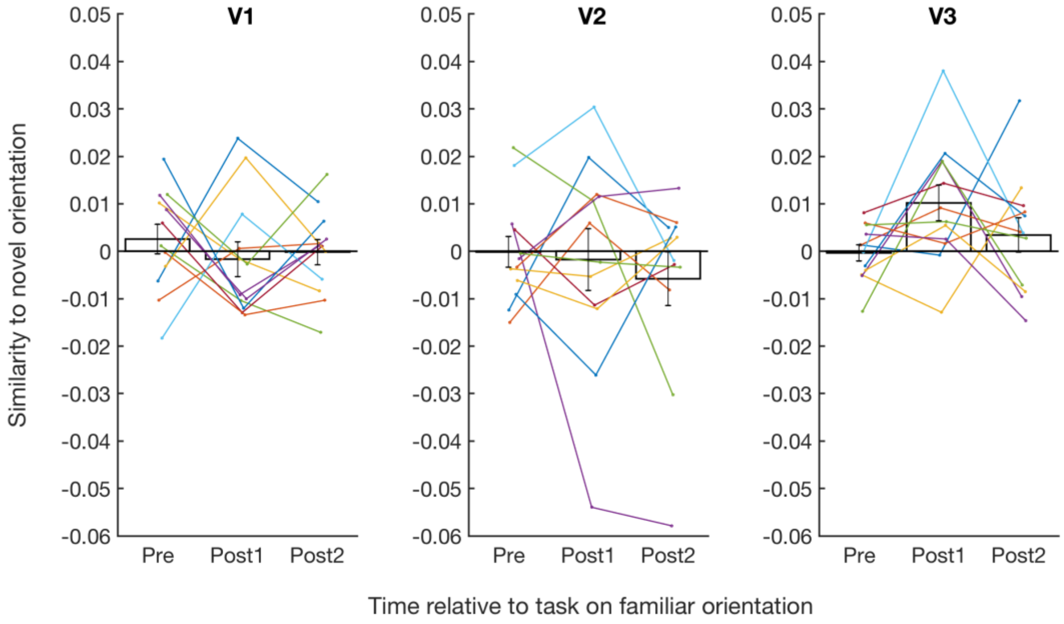

Exposure to the familiar orientation did not induce reactivation of the novel orientation. Indeed, two-way repeated measures ANOVAs with factors time (pre vs. post1 vs. post2) and region (V1, V2, V3) showed no significant main effect or interaction (all P values > 0.1; main effect of time: F(2,22)=0.365, P=0.698, partial η^2=0.032; main effect of region: F(2,22)=2.021, P=0.156, partial η^2=0.155; interaction between time and region: F(4,44)=1.361, P=0.263, partial η^2=0.110). 'Pre' refers to two initial scans before subjects saw any stimuli (5 min/scan; combined into a single 'Pre' baseline). 'Post1' and 'Post2' refer to the first and the second post-task scans immediately after exposure to the familiar orientation. Error bars indicate s.e.m. N=12.

Supplementary Fig. 4. No awake reactivation after exposure to the novel orientation in areas outside V1-V3.

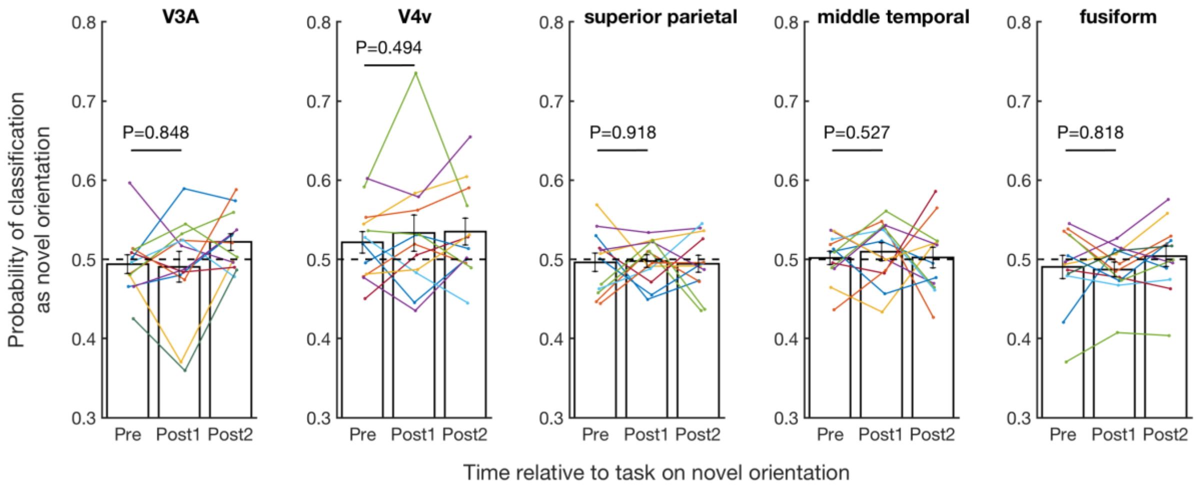

We explored whether awake reactivation occurs outside of the early visual areas in the context of our stimuli. To do so, we created ROIs for V3A, V4v, superior parietal cortex, middle temporal cortex and fusiform cortex, which are the regions found to contain decodable information about the stimulus identity. However, brain activity was not more likely to be classified as the novel orientation shortly after exposure to the novel orientation in any of these areas. Indeed, one-way repeated measures ANOVAs with a factor time (pre vs. post1 vs. post2) showed no significant main effect of time in any of these ROIs (all P values > 0.1; V3A: F(2,22)=1.981, P=0.162, partial η^2=0.153; V4v: F(2,22)=0.393, P=0.680, partial η^2=0.034; superior parietal cortex: F(2,22)=0.029, P=0.971, partial η^2=0.003; middle temporal cortex: F(2,22)=0.122, P=0.886, partial η^2=0.011; fusiform cortex: F(2,22)=1.264, P=0.302, partial η^2=0.103). Furthermore, none of these ROIs exhibited significant changes between the pre and post1 periods (all P values > 0.4; V3A: T(11)=0.196, P=0.848, Hedges' g=0.057; V4v: T(11)=-0.707, P=0.494, Hedges' g=0.168; superior parietal cortex: T(11)=-0.106, P=0.918, Hedges' g=0.043;

middle temporal cortex: T(11)=-0.653, P=0.527, Hedges' g=0.203; fusiform cortex: T(11)=0.235, P=0.818, Hedges' g=0.066; paired t-tests). 'Pre' refers to two initial scans before subjects saw any stimuli (5 min/scan; combined into a single 'Pre' baseline). 'Post1' and 'Post2' refer to the first and the second post-task scans immediately after exposure to the novel orientation. The P values in the figure refer to the results of paired t-tests between pre and post1. Error bars indicate s.e.m. N=12.

Supplementary Fig. 5. No awake suppression after exposure to the familiar orientation in areas outside V1-V3.

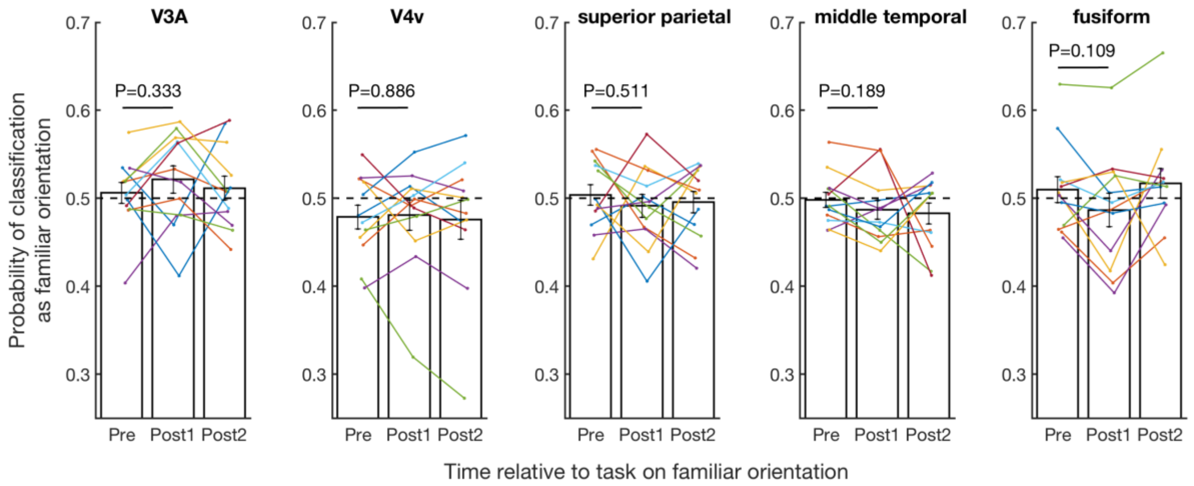

We explored whether awake suppression occurs outside of the early visual areas in the context of our stimuli. To do so, we created ROIs for V3A, V4v, superior parietal cortex, middle temporal cortex and fusiform cortex, which are the regions found to contain decodable information about the stimulus identity. However, brain activity was not any less likely to be classified as the familiar orientation shortly after exposure to the familiar orientation in any of these areas. Indeed, one-way repeated measures ANOVAs with a factor time (pre vs. post1 vs. post2) showed no significant main effect of time in any of these ROIs (all P values > 0.1; V3A: $F(2,22)=0.457$, $P=0.639$, partial $\eta^2=0.040$; V4v: $F(2,22)=0.068$, Huynh-Feldt correction, $P=0.883$, partial $\eta^2=0.006$; superior parietal cortex: $F(2,22)=0.289$, $P=0.752$, partial $\eta^2=0.026$; middle temporal cortex: $F(2,22)=0.557$, Huynh-Feldt correction, $P=0.521$, partial $\eta^2=0.048$; fusiform cortex: $F(2,22)=2.215$, $P=0.133$, partial $\eta^2=0.168$). Furthermore, none of these ROIs exhibited significant changes between the pre and post1 periods (all P values > 0.1; V3A: $T(11)=-1.012$, $P=0.333$, Hedges' $g=0.297$; V4v: $T(11)=-0.147$, $P=0.886$, Hedges' $g=0.036$; superior parietal

cortex: T(11)=0.679, P=0.511, Hedges' g=0.270; middle temporal cortex: T(11)=1.399, P=0.189, Hedges' g=0.312; fusiform cortex: T(11)=1.745, P=0.109, Hedges' g=0.364; paired t-tests). 'Pre' refers to two initial scans before subjects saw any stimuli (5 min/scan; combined into a single 'Pre' baseline). 'Post1' and 'Post2' refer to the first and the second post-task scans immediately after exposure to the familiar orientation. The P values in the figure refer to the results of paired t-tests between pre and post1. Error bars indicate s.e.m. N=12.

Supplementary Table 1. Behavioral and neural measures for newly recruited and revisited subjects.

	**Revisited subjects**	**New subjects**	**P value**
Performance improvement for novel orientation	0.124 ± 0.060	0.090 ± 0.087	0.749
Performance improvement for familiar orientation	0.058 ± 0.078	0.179 ± 0.107	0.382
Awake reactivation at post1 in V1	0.533 ± 0.007	0.524 ± 0.016	0.626
Awake suppression at post1 in V1-V3	0.464 ± 0.022	0.486 ± 0.032	0.578

The two groups did not show any difference in the behavioral and neural effects. The numbers reported for each group are mean ± s.e.m. The P values in the table refer to the results of independent sample t-test between two groups. Unequal variances t-test was applied to reactivation at post1 in V1. N=6 for each group.
