# Supplementary material for: Awake suppression after brief exposure to a familiar stimulus
Source: Commun Biol. 2021 Mar 17;4:348. doi: 10.1038/s42003-021-01863-2 (PMC7969731; doi:10.1038/s42003-021-01863-2)
Supplement: Supplementary file 3 — Reporting Summary [file 42003_2021_1863_MOESM3_ESM.pdf]

nature research

Corresponding author(s): Ji Won Bang

Last updated by author(s): Feb 3, 2021

Reporting Summary

Nature Research wishes to improve the reproducibility of the work that we publish. This form provides structure for consistency and transparency in reporting. For further information on Nature Research policies, see our Editorial Policies and the Editorial Policy Checklist.

Statistics

For all statistical analyses, confirm that the following items are present in the figure legend, table legend, main text, or Methods section.

n/a	Confirmed	
☐	☒	The exact sample size (*n*) for each experimental group/condition, given as a discrete number and unit of measurement
☐	☒	A statement on whether measurements were taken from distinct samples or whether the same sample was measured repeatedly
☐	☒	The statistical test(s) used AND whether they are one- or two-sided<br>*Only common tests should be described solely by name; describe more complex techniques in the Methods section.*
☒	☐	A description of all covariates tested
☐	☒	A description of any assumptions or corrections, such as tests of normality and adjustment for multiple comparisons
☐	☒	A full description of the statistical parameters including central tendency (e.g. means) or other basic estimates (e.g. regression coefficient) AND variation (e.g. standard deviation) or associated estimates of uncertainty (e.g. confidence intervals)
☐	☒	For null hypothesis testing, the test statistic (e.g. F, t, r) with confidence intervals, effect sizes, degrees of freedom and P value noted<br>*Give P values as exact values whenever suitable.*
☒	☐	For Bayesian analysis, information on the choice of priors and Markov chain Monte Carlo settings
☒	☐	For hierarchical and complex designs, identification of the appropriate level for tests and full reporting of outcomes
☐	☒	Estimates of effect sizes (e.g. Cohen's *d*, Pearson's *r*), indicating how they were calculated

Our web collection on statistics for biologists contains articles on many of the points above.

Software and code

Policy information about availability of computer code

Data collection	We used Psychophysics Toolbox 3.
Data analysis	We used Freesurfer (version 6.0) and Sparse Logistic Regression Toolbox (SLR toolbox version 1.2.1alpha; http://www.cns.atr.jp/~oyamashi/SLR_WEB.html).

For manuscripts utilizing custom algorithms or software that are central to the research but not yet described in published literature, software must be made available to editors and reviewers. We strongly encourage code deposition in a community repository (e.g. GitHub). See the Nature Research guidelines for submitting code & software for further information.

Data

Policy information about availability of data

All manuscripts must include a data availability statement. This statement should provide the following information, where applicable:

- Accession codes, unique identifiers, or web links for publicly available datasets
- A list of figures that have associated raw data
- A description of any restrictions on data availability

Data for Figures 2-4 and Supplementary Figures 1-5 are freely available at https://osf.io/kmrwf/

Field-specific reporting

Life sciences study design

All studies must disclose on these points even when the disclosure is negative.

Sample size	Sample size (N=12) was determined based on previous studies on visual perceptual learning that involve multi-day designs (Adini et al., 2002; Jeter et al., 2010; Qu et al., 2010; Rokem and Silver, 2010; Baldassarre et al., 2012; Jehee et al., 2012; Guidotti et al., 2015; Shibata et al., 2016; Bang et al., 2018; Zhang and Tadin, 2019).
Data exclusions	No data were excluded from the analyses.
Replication	N/A
Randomization	Randomization was not relevant because the current study had 1 experimental group.
Blinding	Blinding was not relevant because the current study had 1 experimental group.

Reporting for specific materials, systems and methods

We require information from authors about some types of materials, experimental systems and methods used in many studies. Here, indicate whether each material, system or method listed is relevant to your study. If you are not sure if a list item applies to your research, read the appropriate section before selecting a response.

Materials & experimental systems

n/a	Involved in the study	
☒	☐	Antibodies
☒	☐	Eukaryotic cell lines
☒	☐	Palaeontology and archaeology
☒	☐	Animals and other organisms
☐	☒	Human research participants
☒	☐	Clinical data
☒	☐	Dual use research of concern

Methods

n/a	Involved in the study	
☒	☐	ChIP-seq
☒	☐	Flow cytometry
☐	☒	MRI-based neuroimaging

Human research participants

Policy information about studies involving human research participants

Population characteristics	Twelve subjects (19-27 years old, 5 females) participated in this study. All subjects had normal or corrected-to-normal vision and did not have any history of neurological disorders.
Recruitment	Participants were recruited from students at Georgia Institute of Technology. We did not have any self-selection bias.
Ethics oversight	The study was approved by Institutional Review Board of Georgia Institute of Technology.

Note that full information on the approval of the study protocol must also be provided in the manuscript.

Magnetic resonance imaging

Experimental design

Design type	task
Design specifications	Decoder construction scan: The decoder construction scan had 10 runs (1 run = 300 s) each consisting of 18 trials (1 trial = 16 s) with two fixation periods (each 6 s) at the beginning and end of the run. Each trial consisted of a 12-s stimulus presentation period and a 4-s response period.<br>Pre-task and post-task scans: The pre- and post-task scans consisted of two 5-min scans each. The purpose of the pre- and post-task scans was to record subjects' spontaneous brain activity before and after the task.
Behavioral performance measures	We recorded the button response and the reaction time. To make sure that subjects performed the task as expected, we used staircase procedure in the orientation detection task and the frequency detection task. We monitored whether the step size reduced and stimulus intensity converged after trials.

Acquisition

Imaging type(s)	Structural and functional images
Field strength	3 Tesla
Sequence & imaging parameters	Anatomical images were obtained using a T1-weighted MPRAGE sequence (256 slices, voxel size = 1 x 1 x 1 mm, TR = 2530 ms, FOV = 256 mm). Functional images were collected using a gradient echo-planar imaging sequence (33 slices, voxel size = 3 x 3 x 3.5 mm, TR = 2000 ms, TE = 30 ms, flip angle = 79 degree). The slices covered the whole brain and were parallel to the AC-PC plane.
Area of acquisition	A whole brain

Diffusion MRI ☐ Used ☒ Not used

Preprocessing

Preprocessing software	The imaging data were preprocessed using Freesurfer version 6.0. We performed motion correction and intensity normalization (z score). We did not perform spatial and temporal smoothing for the purpose of multivoxel pattern analysis.
Normalization	We normalized (z scored) each voxel's BOLD time courses within each run.
Normalization template	We analyzed the data in the native subject space.
Noise and artifact removal	We removed voxels that had spikes greater than 10 SDs from the mean and removed a linear trend in the BOLD time course.
Volume censoring	We computed framewise displacement (FD) using the formula from Power et al. (2012). Our criteria was FD > 0.5. We found that only 2 volumes out of a total of 10800 volumes had FD greater than 0.5 (0.019% of all volumes). These 2 volumes did not have any effect on the results.

Statistical modeling & inference

Model type and settings	We used linear sparse logistic regression for multivoxel pattern analysis. This method selects the relevant voxels in the ROIs and calculates their weights for classification.
Effect(s) tested	We tested the probability that neural patterns are classified as either novel or familiar orientation using ANOVAs. We also tested how similar the multivoxel patterns are to the template patterns of each orientation using ANOVAs.

Specify type of analysis: ☐ Whole brain ☒ ROI-based ☐ Both

Anatomical location(s)	We delineated V1, V2, V3, V3A, and ventral V4 using a standard retinotopy methods (Sereno et al., 1995; Tootell et al., 1997). Other 27 ROIs covering a whole brain were obtained using Freesurfer's cortical parcellation method.
Statistic type for inference (See Eklund et al. 2016)	Voxel-wise
Correction	In the post-hoc tests of ANOVAs, we used Bonferroni correction.

Models & analysis

n/a	Involved in the study
☒	☐ Functional and/or effective connectivity
☒	☐ Graph analysis
☐	☒ Multivariate modeling or predictive analysis

Multivariate modeling and predictive analysis	We used linear sparse logistic regression for multivoxel pattern analysis. This method selects the relevant voxels in the ROIs and calculates their weights for classification. We trained the decoder to classify the brain activity patterns in each ROI to either 45 or 135 using the 180 data samples. To verify the robustness of the decoder, we tested its reliability using a 10-fold cross-validation where the decoder was re-trained on nine runs and tested on the remaining run.<br>In the additional pattern similarity analysis, we constructed multivoxel template patterns for each orientation and then computed how similar the brain activity patterns during the pre- and post-task scans are to the template patterns of each orientation using Pearson correlation.
